# Consensus Definition of Nonallergic Rhinopathy, Previously Referred to as Vasomotor Rhinitis, Nonallergic Rhinitis, and/or Idiopathic Rhinitis

**DOI:** 10.1097/WOX.0b013e3181a8e15a

**Published:** 2009-06-15

**Authors:** Michael A Kaliner, James N Baraniuk, Michael Benninger, Jonathan A Bernstein, Phil Lieberman, Eli O Meltzer, Robert M Naclerio, Russell A Settipane, Judith R Farrar

**Affiliations:** 1 Institute for Asthma and Allergy, Chevy Chase, Md; 2 LifeSciences Press, Washington, DC; 3 George Washington University School of Medicine, Washington, DC; 4 Division of Rheumatology, Immunology and Allergy, Georgetown University, Washington, DC; 5 Head and Neck Institute, Cleveland, OH; 6 University of Cincinnati College of Medicine, Cincinnati, Ohio; 7 Division of Allergy and Immunology, Departments of Medicine and Pediatrics, University of Tennessee, Germantown, TN; 8 Allergy & Asthma Medical Group & Research Center, University of California, San Diego, CA; 9 Section of Otolaryngology-Head and Neck Surgery, University of Chicago, Chicago, IL; 10 Allergy & Asthma Center, Brown Medical School, Providence, RI

**Keywords:** nonallergic vasosmotor rhinitis, nonallergic rhinitis, vasomotor rhinitis, idiopathic rhinitis, nonallergic rhinopathy

## Abstract

"Nonallergic vasomotor rhinitis" (also referred to as nonallergic rhinitis and/or idiopathic rhinitis) is a term that has been used to describe a common nasal condition of unclear pathophysiology. The lack of straightforward diagnostic criteria is limiting; research for better treatment options requires the definition of homogeneous populations characterized by well-defined inclusion and exclusion criteria. Following considerable discussion and counterpoints at a roundtable conference convened in December 2008, we proposed to change the terminology to reference this condition as "nonallergic rhinopathy." Nonallergic rhinopathy is a chronic nasal condition with symptoms that may be perennial, persistent, intermittent, or seasonal and/or elicited by recognized triggers. There is a well-recognized set of clinical exposures that lead to the symptoms, predominantly congestion and rhinorrhea. The clinical characteristics as outlined provide well-defined inclusion and exclusion criteria that should permit precise identification of patients for participation in clinical trials.

## 

The papers presented in the first part of these proceedings were delivered at the roundtable meeting (December 13, 2008, Washington, DC) with the intent of reaching a consensus definition of nonallergic rhinopathy (formerly referred to as vasomotor rhinitis, nonallergic rhinitis, and/or idiopathic rhinitis). Consensus was attained following considerable discussion and counterpoints by all participants.

I. "Nonallergic rhinopathy" (NAR) is recommended to replace the term vasomotor rhinitis (VMR). VMR suggests that *intrinsic *nasal vascular and glandular abnormalities are the principle physiological causes of inflammation of the nasal mucous membrane. However, current information suggests that NAR is probably due to neurosensory abnormalities and does not include inflammation as an important component. Thus, we believe that it is more accurate to term this condition a *rhinopathy *(a disorder of the nose) rather than a form of rhinitis (inflammation of the nose). The single unequivocal criterion for these patients is that they are not allergic: *nonallergic rhinopathy *is a more appropriate term for this disorder.

II. NAR is defined by clinical characteristics, which are summarized below and described in detail in the series of related articles from the consensus conference.

A. NAR is a chronic disease with some, but not necessarily all, of the following symptoms:

1. Primary symptoms:

a. Nasal congestion.

b. Rhinorrhea.

2. Other associated symptoms:

a. Postnasal drip in the absence of a pharyngeal cause of mucus hypersecretion or acid reflux disease.

b. Throat clearing.

c. Cough.

d. Eustachian tube dysfunction (ear pressure/popping/pain).

e. Sneezing.

f. Hyposmia.

g. Facial pressure/headache.

B. Symptoms of NAR may be perennial, persistent, or seasonal (i.e., climatic-see below) and/or elicited by defined triggers. These triggers may include the following:

1. Cold air.

2. Changes in climate (such as temperature, humidity, and barometric pressure).

3. Strong smells (such as perfume, cooking smells, flowers, and chemical odors).

4. Environmental tobacco smoke.

5. Changes in sexual hormone levels.

6. Pollutants and chemicals (e.g., volatile organics).

7. Exercise.

8. Alcohol ingestion.

Symptoms may be described as perennial, persistent, intermittent, or seasonal and occur in response to climatic shifts in temperature, humidity, and barometric pressure. A patient's symptoms may be brought on by 1 or more of the defined precipitants. There are no current data indicating that patients responsive to environmental climate changes as a trigger differ from those triggered by perfumes or strong smells. Moreover, there is no current information suggesting that a patient with this set of clinical symptoms and characteristics, and for whom no triggers are identified, differ from patients with clearly defined triggers. Thus, NAR may be diagnosed regardless of the presence or the absence of defined triggers.

C. There is a female-to-male incidence ratio for NAR of 2:1 to 3:1.

D. NAR presents predominantly with adult onset.

E. The nasal mucosa in NAR usually appears normal, but may sometimes appear red and beefy with scant mucus.

F. NAR is associated with negative or irrelevant skin prick tests or antigen-specific IgE tests (formerly referred to as radioallergosorbent tests).

G. NAR may present with concomitant conditions such as the following:

1. Food-related rhinorrhea.

2. Mild nasal eosinophilia (< 5%).

3. Eustachian tube dysfunction (ear pressure/popping/pain).

4. Senile rhinitis.

H. NAR symptoms are not caused by other known etiological factors for rhinopathy, such as the following:

1. Chronic rhinosinusitis or nasal polyps.

2. Nonallergic rhinitis with eosinophilia syndrome with nasal eosinophilia > 5%.

3. Aspirin-related chronic rhinosinusitis, nasal polyps, or asthma, although NAR is usually seen as one of the clinical characteristics of aspirin-exacerbated respiratory disease.

4. Infectious rhinitis or rhinosinusitis (eg, viral upper respiratory infections, bacterial/fungal rhinosinusitis, and bacterial rhinitis).

5. Anatomical abnormalities.

6. Drug usage (e.g., adverse effect of systemic medication and excess use of topical decongestants).

7. Cerebrospinal fluid leak.

8. Pregnancy.

III. In summary, nonallergic rhinopathy is a chronic condition with symptoms that may be perennial and/or elicited by recognized triggers. The clinical characteristics provide well-defined inclusion and exclusion criteria that should permit precise identification of patients with this disease category for further study. The next issue of this journal will include our recommendations for those inclusion and exclusion criteria based on the in-depth discussion and counterpoints at the roundtable meeting.

## Note

Dr. Baraniuk has nothing to disclose.

Dr. Benninger discloses that he has received grant/research support from GlaxoSmithKline (GSK). He is a consultant, or on an advisory board or the speakers bureau for GSK, Alcon, Schering Plough, Sanofi-Aventis, and Greer.

Dr. Bernstein discloses that he has received grant/research support from Merck, AstraZeneca, Teva Pharmaceuticals, Meda Pharmaceuticals, and Sepracor, among other companies.

Dr. Farrar has nothing to disclose.

Dr. Kaliner discloses that he has received grant/research support from GlaxoSmithKline (GSK), Sanofi-Aventis Pharmaceuticals, Schering Plough Corporation, Pfizer Inc, Meda Pharmaceuticals, AstraZeneca, Merck, Alcon Laboratories, among others. He is a consultant, or on an advisory board or the speakers bureau for GSK, Novartis, Genentech, Alcon, Meda, Cornerstone, Schering, Sepracor, and Sanofi-Aventis, among others.

Dr. Lieberman discloses that he is a consultant, or on an advisory board or the speakers bureau for GlaxoSmithKline (GSK), Sanofi-Aventis Pharmaceuticals, Schering Plough Corporation, Meda Pharmaceuticals, and Alcon Laboratories.

Dr. Meltzer discloses that he has received grant/research support from Alcon, Amgen. Apotex. AstraZeneca, Boehringer Ingelheim, Capnia, Genentech, GlaxoSmithKline, MAP Pharmaceuticals, Meda, Merck, Novartis, Pharmaxis. SanofiAventis, Schering-Plough, Sepracor, Skye Pharma, Teva, Vocel, and Wyeth. He is a consultant, or on an advisory board or the speakers bureau for Abbott, Alcon. Amgen, AstraZeneca, Capnia, Dey Labs, and Genentech.

Dr. Naclerio discloses that he has received grant/research support from GlaxoSmithKline (GSK), Schering Plough Corporation, and Merck. He also is a consultant, or on an advisory board or the speakers bureau for those companies.

Dr. Settipane discloses that he has received grant/research support from GlaxoSmithKline (GSK), Sanofi-Aventis Pharmaceuticals, Meda Pharmaceuticals, Genenetech/Novartis, and Alcon Laboratories. He is a consultant, or on an advisory board or the speakers bureau for Glaxo-SmithKline (GSK), Sanofi-Aventis Pharmaceuticals, Genenetech/Novartis, and Alcon Laboratories.

Presented at a roundtable conference held in December 2008 in Washington, DC. The meeting was sponsored by the TREAT Foundation (Washington, DC) and supported by an unrestricted educational grant from Meda Pharmaceuticals. The funding company did not have any input into the development of the meeting or the proceedings; the company was not represented at the roundtable meeting.

